# Expression of neutrophil gelatinase-associated lipocalin (NGAL) in the gut in Crohn’s disease

**DOI:** 10.1007/s00441-018-2860-8

**Published:** 2018-06-05

**Authors:** Silje Thorsvik, Ingunn Bakke, Atle van Beelen Granlund, Elin Synnøve Røyset, Jan Kristian Damås, Ann Elisabet Østvik, Arne Kristian Sandvik

**Affiliations:** 1 Centre of Molecular Inflammation Research, NTNU, Norwegian University of Science and Technology, Trondheim, Norway; 2 Department of Clinical and Molecular Medicine, NTNU, Norwegian University of Science and Technology, 7489 Trondheim, Norway; 3 Department of Gastroenterology, St Olav’s University Hospital, Trondheim, Norway; 4 Clinic of Medicine, St Olav’s University Hospital, Trondheim, Norway; 5 Department of Pathology, St Olav’s University Hospital, Trondheim, Norway; 6 Department of Infectious Diseases, St Olav’s University Hospital, Trondheim, Norway

**Keywords:** LCN2, Inflammatory bowel disease, Crohn’s disease, Paneth cells, Enteroendocrine cells

## Abstract

The antimicrobial glycoprotein neutrophil gelatinase-associated lipocalin (NGAL) is strongly expressed in several infectious, inflammatory and malignant disorders, among these inflammatory bowel disease (IBD). Fecal and serum NGAL is elevated during active IBD and we have recently shown that fecal NGAL is a novel biomarker for IBD with a test performance comparable to the established fecal biomarker calprotectin. This study examines expression of NGAL in the healthy gut and in Crohn’s disease (CD), with emphasis on the previously unexplored small intestine. Pinch biopsies were taken from active and inactive CD in jejunum, ileum and colon and from the same sites in healthy controls. Microarray gene expression showed that the NGAL gene, *LCN2*, was the second most upregulated among 1820 differentially expressed genes in terminal ileum comparing active CD and controls (FC 5.86, *p* = 0.027). Based on immunohistochemistry and in situ hybridization findings, this upregulation most likely represented increased expression in epithelial cells. Double immunofluorescence showed NGAL expression in 49% (range 19–70) of Paneth cells (PCs) in control ileum with no change during inflammation. In healthy jejunum, the NGAL expression in PCs was weak to none but markedly increased during active CD. We further found NGAL also in metaplastic PCs in colon. Finally, we show for the first time that NGAL is expressed in enteroendocrine cells in small intestine as well as in colon.

## Introduction

Neutrophil gelatinase-associated lipocalin (NGAL, also known as lipocalin 2 or siderocalin, gene symbol *LCN2*) was first discovered in 1996. This 24 kDa glycoprotein is part of the lipocalin superfamily known to bind and transport small hydrophobic molecules. NGAL was originally shown to be a component of specific granules in human neutrophils (Kjeldsen et al. 2000) and has been identified with cell-type-specific expression in several tissues like bronchus, stomach, small intestine, pancreas, kidney, prostate gland and thymus (Friedl et al. 1999).

NGAL is a potent bacteriostatic agent, acting by binding bacterial catecholate-type ferric siderophores (Flo et al. 2004; Goetz et al. 2002; Miethke and Skerra 2010). However, NGAL is also present in several aseptic inflammatory disorders, as well as in various cancer types. Bao et al. found evidence for catechol as an endogenous siderophore and demonstrated that the catechol-NGAL complex plays a role in trafficking iron in non-infectious tissue (Bao et al. 2010). NGAL has also been suggested to act as a growth and differentiation factor (Chakraborty et al. 2012; Devireddy et al. 2005; Schmidt-Ott et al. 2007) and as an adipokine (Wang et al. 2007; Yan et al. 2007). It has further been suggested to have chemotactic properties, to act as an acute phase protein and to stabilize the proteolytic enzyme MMP-9 (Chakraborty et al. 2012). A recent study suggested a role as an appetite regulating hormone (Mosialou et al. 2017). NGAL can be induced by various inflammatory factors, among these toll-like receptor 3 and 5 and cytokines like TNF, IL1β, IL22 and IL17 (Nielsen et al. 1996; Ostvik et al. 2013; Singh et al. 2016; Stallhofer et al. 2015). NGAL has been found to be a promising biomarker for acute kidney injury (Mishra et al. 2005).

The present study is relevant to the ongoing exploration of NGAL as a biomarker for gastrointestinal disease, in particular the two forms of inflammatory bowel disease (IBD), ulcerative colitis (UC) and Crohn’s disease (CD). While UC is characterized by inflammation of the colonic mucosa, CD is a deeper, transmural inflammation that can affect all parts of the gastrointestinal tract, with the predilection area being the terminal ileum. Nielsen et al. and our group have shown that the gene encoding NGAL, *LCN2*, is one of the most upregulated genes in colonic biopsies from patients with UC or CD compared to healthy controls (Nielsen et al. 1996; Ostvik et al. 2013). Immunohistochemistry (IHC) shows strong expression of NGAL in the human colonic epithelial cells and in mucosal granulocytes during active inflammation (Ostvik et al. 2013).

NGAL is increased in serum and feces during active IBD and several studies have explored its role as a disease biomarker (de Bruyn et al. 2015; de Bruyn et al. 2014; Janas et al. 2014; Magro et al. 2017; Stallhofer et al. 2015; Yesil et al. 2013). Our group found fecal NGAL to be massively increased in active IBD, with a test performance comparable to the established fecal biomarker calprotectin (Thorsvik et al. 2017). Moreover, we observed that NGAL is easily detectable in feces from most healthy controls, despite weak to no expression of NGAL in the colonic mucosa. Most likely, this NGAL derives from the small intestine, where its expression in health and disease has not been studied. Thus, in our ongoing studies of NGAL as a biomarker for IBD, we found it important to characterize NGAL expression also in the small intestine, in particular the ileum, which is a predilection area for CD. To this end, we investigated the expression of NGAL in healthy individuals and in patients with small intestinal CD using gene expression analysis, IHC and in situ hybridization (ISH).

The small intestinal epithelium consists of various cell types with different functions (Peterson and Artis 2014). In the present study, we show NGAL expression in Paneth cells (PCs) and enteroendocrine cells (EECs). PCs are located in the crypt bottoms of the small intestine. These are pyramidally shaped columnar cells rich in granules intermingled between LGR5-positive stem cells in the crypts of Lieberkühn (Bevins and Salzman 2011). PCs are rare in the healthy colon but are commonly seen as metaplastic PCs in IBD patients. The antimicrobial substances defensin-5, defensin-6 and lysozyme are common markers for PCs. EECs typically have an apical cytoplasmic process with microvilli that extend towards the luminal surface. Various markers exist for EECs, among these chromogranin A, which is considered a general EEC marker (Gunawardene et al. 2011).

## Materials and methods

### Samples

Patients with known CD and healthy controls coming for an ileo-colonoscopy or enteroscopy at the Department of Gastroenterology and Hepatology, St. Olav’s University Hospital, Trondheim, Norway, were included in the study after written informed consent. The study was approved by the Regional Committee for Medical and Health Research Ethics, approval nos. 5.2007.910 and 2013/212/REKmidt.

Pinch biopsies were obtained during the ileo-colonoscopy from the terminal ileum or from the neo-terminal ileum in patients having undergone ileocecal resection. Two of the biopsies from each patient were formalin-fixed for IHC and ISH and two were snap-frozen for gene expression analysis. A gastrointestinal pathologist (ESR) classified the biopsies into normal, chronic active, or chronic inactive inflammation and samples with discrepancy between endoscopic and histologic diagnoses were excluded. The patients with chronic inactive inflammation all previously had verified CD of the terminal ileum. Patients coming for an endoscopy for other reasons than IBD and with normal endoscopic and histological findings served as controls. For comparison of PCs between proximal and distal small intestine and for comparison between active jejunal CD and controls, biopsies from the jejunum were obtained by enteroscopy. Moreover, for comparison with metaplastic PCs of the colon, biopsies from patients with active colonic IBD were obtained from a previously described biobank (Granlund et al. 2013).

### Microarray

Biopsies from six individuals of each of the categories active CD, inactive CD and healthy controls were analyzed for gene expression using microarray. Patient characteristics are given in Table 1. Microarray gene expression analysis followed standard protocols. In brief, cRNA was prepared with Ambion’s Illumina® TotalPrep™ RNA Amplification Kit (Thermo Fisher Scientific, cat. no. AMIL1791), using 300 ng total RNA as input material. For each sample, the biotin-labeled cRNA concentrations were determined (NanoDrop, Thermo Fisher Scientific) and 750 ng hybridized to HumanHT-12 Expression BeadChips, scanned on a Illumina HiScan instrument (Illumina Inc., CA, USA) and processed in GenomeStudio (version 2011.1).

**Table 1:** Characteristics of subjects enrolled in microarray analysis of ileum. *TNFα* tumor necrosis factor alpha, *MAb* monoclonal antibody

		Controls	CD, active	CD, inactive
Number of subjects		6	6	6
Average age, years (range)		41 (26–57)	34 (21–47)	35 (18–62)
Treatment	5-aminosalicylic acid	–	0	1
	Anti-TNFα MAb	–	1	2
	Azathioprine	–	1	1
	Corticosteroids	–	4	1
	None	6	2	3

GenomeStudio output was read and processed using the lumi R-package (Du et al. 2008) and probe annotations were added from the IlluminaHumanv4.db R-package (Dunning et al. 2015). The gene level data values were filtered, log2 transformed and quantile normalized to create the expression values used for statistical modeling. Differential expression was modeled using a linear model and fitted using the limma R-package (Ritchie et al. 2015). Significance was considered on genes with a false discovery adjusted (Benjamini-Hochberg method) *p* value below 0.05.

### Immunohistochemistry and double immunofluorescence

Formalin-fixed, paraffin-embedded (FFPE) biopsies were cut into 4 μm thick sections and mounted on glass slides. The sections were pretreated with permeabilization, quenching of endogenous peroxidases and antigen retrieval. Primary antibodies for human NGAL (Abcam, cat. no. ab41105 and AntiBodyShop, cat. no. ABS 062-14B), DEFA6 (Sigma, cat. no. HPA019462) and CgA (Immunostar, cat.no. 20086) were used with an incubation time of 1 h at room temperature or overnight at 4 °C. Secondary antibody was rabbit/mouse EnVision-HRP and visualization was made with DAB^+^ (Dako, Glostrup, Denmark, cat. no. K5007) before counterstaining with hematoxylin. Double immunofluorescence staining was performed using the MaxDouble™ M488&R650 ImmunoFluorescence Double Staining Kit for human tissue (cat. no. DSMR-H3, MaxVision Biosciences Inc., WA, USA) according to the manufacturer’s instructions, with antigen retrieval in boiling Tris-EDTA buffer (pH 9.0), followed by incubation with the primary antibodies at 4 °C and DAPI as nuclear counterstaining. Omitting the primary antibody (IF and IHC) and matching isotype immunoglobulins (IHC only) were used as negative controls.

### In situ hybridization

ISH was performed using an RNAscope 2.5 HD Reagent Kit (Brown or Duplex) for FFPE tissue (Advanced Cell Diagnostics (ACD), Hayward, CA, USA) and probes for *LCN2*, *CHGA* and *DEFA6* according to the manufacturer’s instructions. All probes were gene- and species-specific. All samples were tested with positive and negative control probes supplied by the manufacturer.

### Quantification of NGAL in Paneth cells and enteroendocrine cells

IHC of NGAL was performed on biopsies from a total of 67 individuals (24 active CD, 14 inactive CD, 29 controls). In some samples, especially in the inflamed biopsies, it could be difficult to distinguish between PC and EEC expression using IHC. Double fluorescence was thus used for comparison of expression of NGAL in the two cell types between patient groups. To compare expression of NGAL in PCs between active ileal CD and controls, five biopsies in each group were double-stained for NGAL and the PC specific Defensin-6 (DEFA6). The number of dual positive cells and the total number of DEFA6-positive cells (89–214 cells per biopsy in active CD and 62–133 in controls) were counted in all biopsies and the percentages of NGAL expressing PCs were calculated. To examine NGAL-expression in EECs, five biopsies from healthy ileum, five from healthy colon and four from active CD (two colon, two ileum) were double-stained with NGAL and chromogranin A (CgA). The number of dual positive cells and the total number of CgA-positive cells (17–40 cells per biopsy in colon and 35–80 in ileum) were counted in all biopsies and the percentage of NGAL expressing EECs was calculated.

For comparison of NGAL expression between proximal and distal small intestine, the ileal controls were compared to four controls from jejunum (61–136 DEFA6-positive cells per biopsy) using the same method as described above. For comparison of *LCN2* mRNA expression between proximal and distal small intestine, ISH was used and the number of *LCN2*-positive PCs was quantified in five biopsies from jejunum and eight from terminal ileum. We used score 0 for no staining, 1 for staining of ≤ 2 PCs per crypt and 2 for > 3 per crypt. Serial sections with staining for the PC-specific DEFA6 were used to confirm the presence of these cells in the biopsies.

For identification of PC metaplasia, colonic biopsies with known active IBD-inflammation were stained with the PC-specific DEFA6. Biopsies from five patients with confirmed PC metaplasia were double-stained for NGAL and DEFA6 and quantified as described above (15–38 DEFA6-positive cells per biopsy).

### Image capture and processing

Chromogenic images were acquired by using a Nikon E400 microscope and NIS-Elements BR imaging software (Nikon Instruments, Melville, NY, USA). Immunofluorescence images were captured using a Leica SP8 inverted microscope (LeicaMicrosystems, Mannheim, Germany) and further processed using ImageJ (Wayne Rasband, National Institutes of Health, USA). Color adjustments were made by Lightroom (Adobe, San Jose, CA, USA). InkScape 0.92 was used for subsequent image orientation and cropping.

### Statistics

A Mann-Whitney test was used in the comparison of NGAL expression in PCs and EECs between active CD and controls and in the comparison of NGAL/*LCN2* expression in PCs between ileum and jejunum. A *p* value < 0.05 (two-sided) was considered statistically significant. Individual values are given as median (range). Calculations were performed using Prism 5 (GraphPad Software, San Diego, CA, USA).

## Results

### *LCN2* mRNA overexpression in ileum

**Fig. 1:**
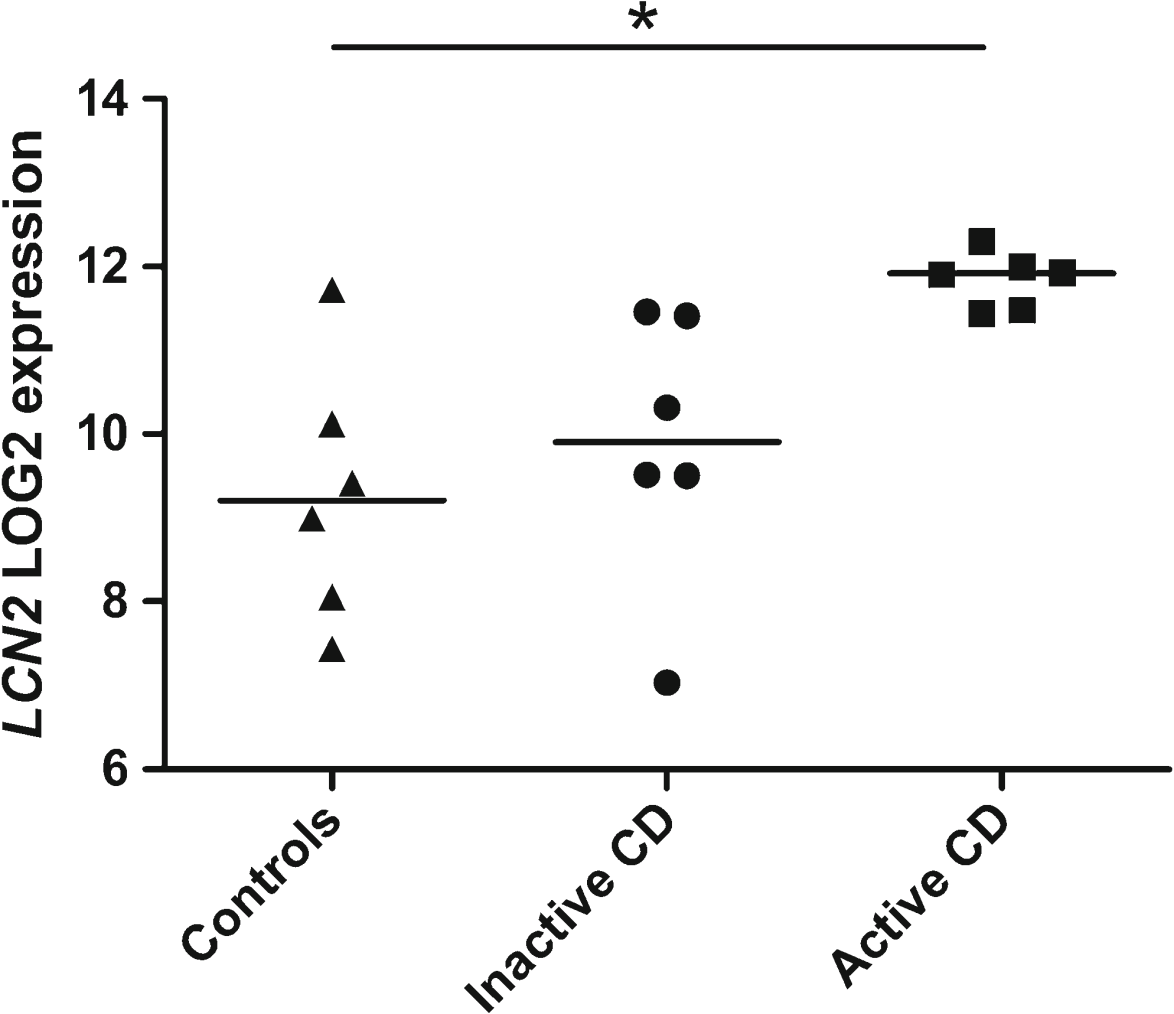
Scatter plot of *LCN2* mRNA levels in microarray gene expression analysis of biopsies from controls, inactive CD and active CD in terminal ileum. *p < 0.05

Microarray gene expression analysis of pinch biopsies from terminal ileum showed that *LCN2* was the second most upregulated gene among all 1820 differentially expressed genes between active CD and controls (fold change (FC) 5.86, *p* = 0.027). Figure 1 shows a scatter plot of *LCN2* expression in the three patient groups. Only *MUC1* was more overexpressed (FC 8.57, *p* = 0.001). When comparing inactive CD with controls, there were no differentially expressed genes. The proinflammatory cytokine IL8 was significantly upregulated (FC 3.23, *p* = 0.03) confirming the inflammatory status of the biopsies from patients with active CD. The 15 most upregulated genes between active CD and controls are listed in Table 2. The whole gene list with the differentially expressed genes is available in Array Express, GSE number E-MTAB-6593.

**Table 2:** List of the 15 most upregulated among 1820 significantly differentially expressed genes in inflamed ileal biopsies vs healthy controls. *N* = 6 in each group. *FC* fold change vs control

Gene symbol	Description	FC	*p* value
MUC1	Mucin-1	8.57	0.001
LCN2	Neutrophil gelatinase-associated lipocalin	5.86	0.027
MMP3	Stromelysin-1	4.50	0.022
CLCA4	Calcium-activated chloride channel regulator 4	4.20	0.050
CXCL9	C-X-C motif chemokine 9	3.71	0.039
NOS2	Nitric oxide synthase, inducible	3.68	0.015
IGFBP5	Insulin-like growth factor-binding protein 5	3.58	0.005
TIMP1	Metalloproteinase inhibitor 1	3.53	0.006
IFITM3	Interferon-induced transmembrane protein 3	3.43	0.002
IFITM2	Interferon-induced transmembrane protein 2	3.25	0.003
ITLN1	Intelectin-1	3.23	0.020
IL8	Interleukin-8	3.23	0.030
S100A9	Protein S100-A9	3.20	0.050
MXRA5	Matrix-remodeling-associated protein 5	3.18	0.001
GPX2	Glutathione peroxidase 2	3.16	0.016

### NGAL-expression in colon, ileum and jejunum

**Fig. 2:**
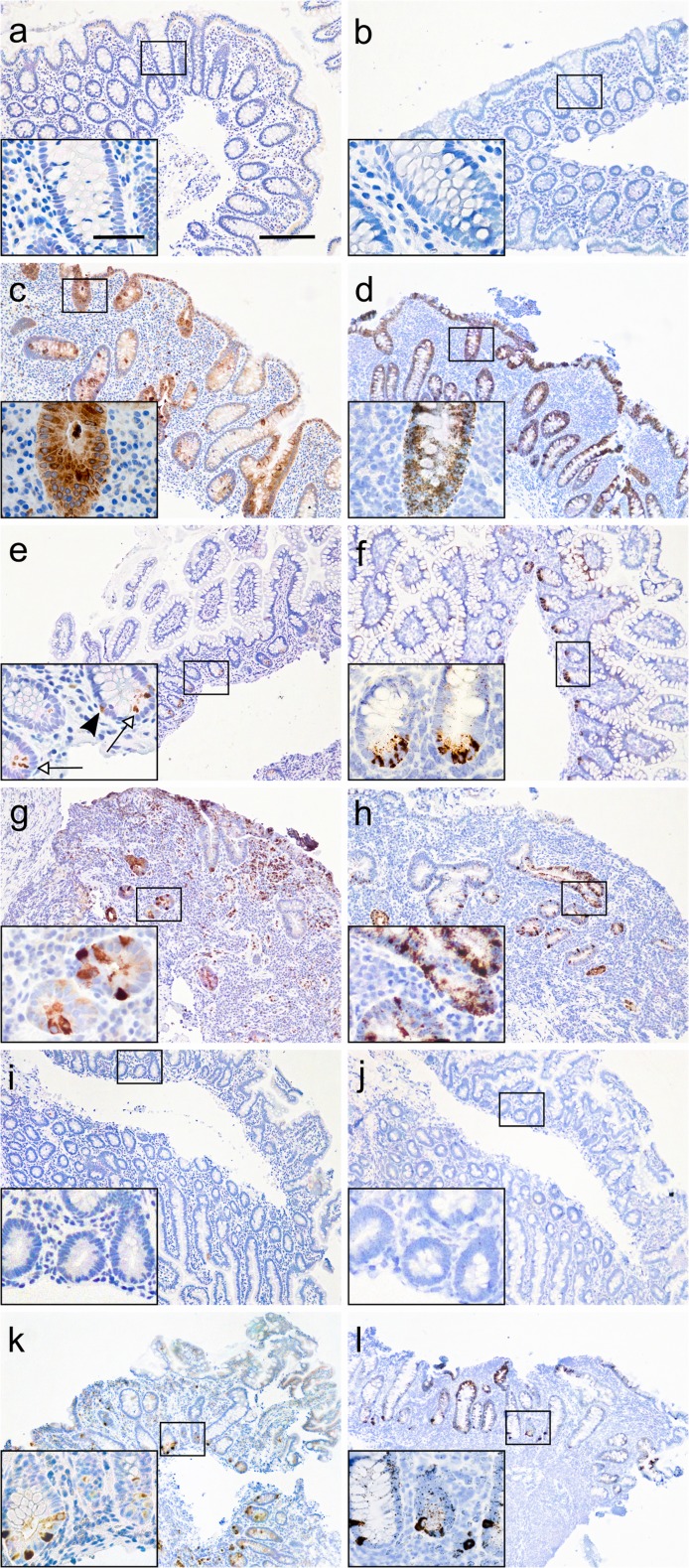
IHC of NGAL and ISH of *LCN2* in biopsies from colon (**a**–**d**), terminal ileum (**e**–**h**) and jejunum (**i**–**l**). **a** IHC of control colon. Weak to no staining of the epithelium. **b** ISH of control colon with no signal. **c** IHC of active CD in colon. Prominent staining of both granulocytes, goblet cells and enterocytes. **d** ISH of active CD in colon with intense signal in the epithelium. **e**, **f** IHC (**e**) and ISH (**f**) of control ileum. Marked expression in PCs (arrows) and EECs (arrowhead). Weak to no staining of the epithelium. **g** IHC of active CD in terminal ileum with markedly distorted architecture and complete loss of villi. Marked staining of granulocytes, the epithelium and scattered PCs and EECs. **h** ISH of active CD in terminal ileum. Expression in epithelium, PCs and EECs. **i**, **j** IHC (**i**) and ISH (**j**) of control jejunum with no expression. **k** IHC of active CD in jejunum with staining of granulocytes, epithelium, PCs and EECs. **l** ISH of active CD in jejunum with expression in the epithelium, PCs and EECs. Scale bar 200 μm (50 μm in inserts)

Figure 2 shows IHC and ISH of NGAL/*LCN2* in the three examined segments of colon, ileum and jejunum in active CD and controls. The results for IHC and ISH for NGAL/*LCN2* in inactive CD were identical to controls (data not shown).

In the colon, consistent with our previous findings (Ostvik et al. 2013), IHC showed intense staining of NGAL in granulocytes, goblet cells and enterocytes of the colonic epithelium in active CD with weak to no staining in the control colons. We now also noted expression in EECs and metaplastic PCs (Fig. 2a, c). ISH confirmed the increased *LCN2* mRNA expression of the inflamed epithelium (Figs. 2b, d), whereas there was no expression in granulocytes. The latter is probably due to prepacking of NGAL in the granulocytes in the bone marrow with *LCN2* mRNA being downregulated in peripheral cells, as shown earlier (Nielsen et al. 1996).

In healthy terminal ileum, there was prominent NGAL/*LCN2* expression in a subset of PCs and EECs, with weak to no expression in epithelial goblet cells or enterocytes (Fig. 2e, f). Inflamed terminal ileum showed expression in granulocytes (IHC only) and epithelial cells, both in crypts and villi. Expression was particularly prominent in biopsies having markedly distorted architecture and loss of villi (Fig. 2g, h). We also noticed a more patchy appearance of the expression in the inflamed ileal epithelium compared to the inflamed colonic epithelium, where expression appeared more continuous.

**Fig. 3:**
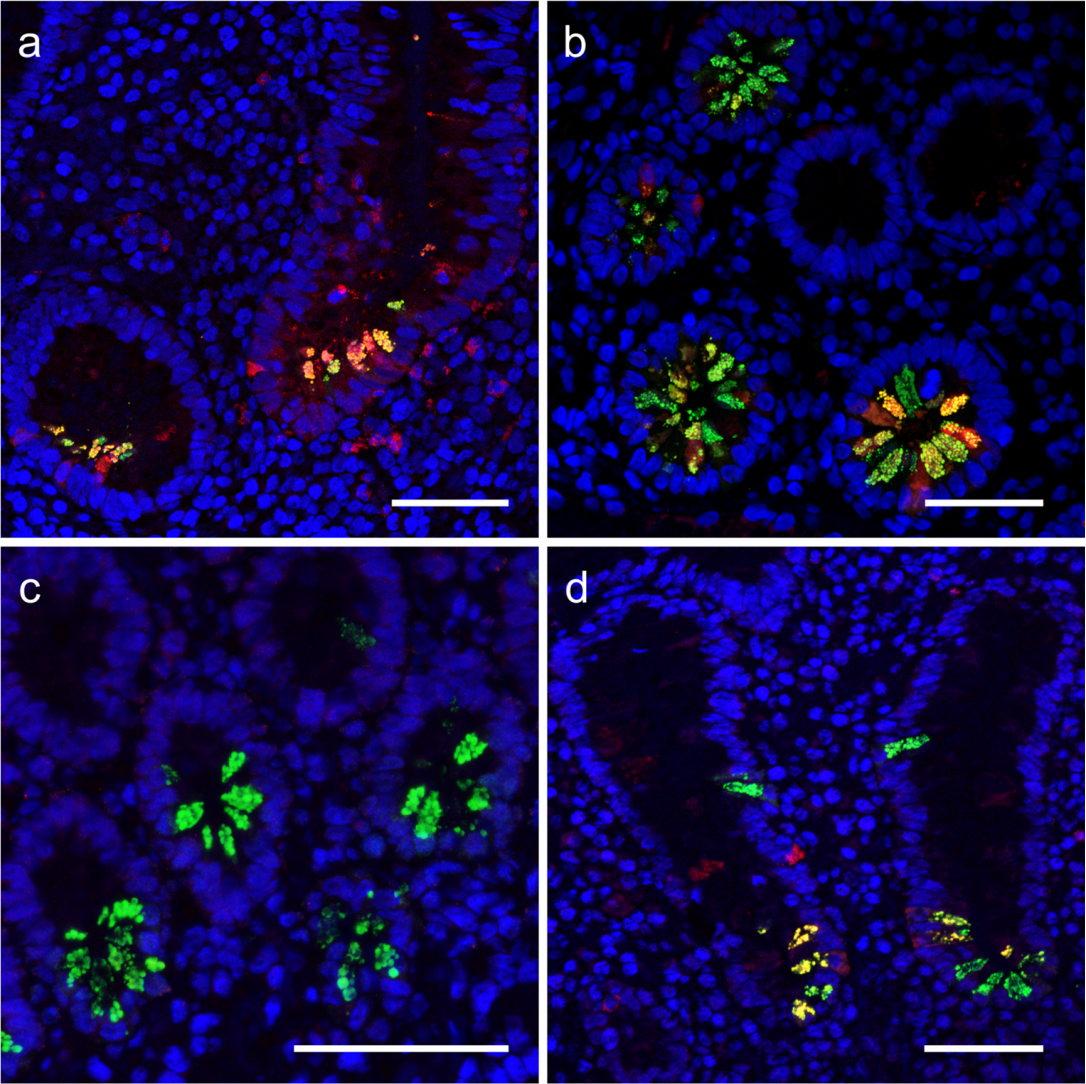
NGAL expression in PCs. Double immunofluorescence. Red: NGAL. Green: DEFA6. Yellow: overlapping signals. **a** Colon. Overlap in a subset of metaplastic PCs in inflamed colon. NGAL expression can also be seen in granulocytes, EECs and in the inflamed epithelium. **b** Terminal ileum. Overlap in a subset of PCs. NGAL also in EECs. **c** No overlap in control jejunum. **d** Overlap in a subset of PCs in inflamed jejunum. NGAL also in EECs. Scale bar 50 μm

In healthy jejunum, there was weak to no NGAL/*LCN2* expression in the epithelium and the EECs. There was low expression in very few PCs (Fig. 2i, j). In inflamed jejunum, however, there was expression in granulocytes (IHC only), epithelial cells, EECs and PCs (Figs. 2k, l and 3d).

### NGAL expression in distinct cell types

#### Paneth cells

In active CD of the colon, we now, in addition to more general epithelial NGAL expression, also noticed prominent expression in granules in scattered crypt bottom cells. Since PC metaplasia is a common finding in the colon during active IBD inflammation, we performed double immunofluorescence staining of NGAL and the PC-specific DEFA6, which confirmed NGAL expression in 56 (40–73) % of metaplastic PCs (Fig. 3a). PC metaplasia can persist for a period after amelioration of inflammation and NGAL staining was positive also in PC metaplasia in inactive CD (data not shown).

Using double immunofluorescence staining, we further assessed the co-localization of NGAL and DEFA6 in ileal PCs. There was a clear overlap between DEFA6 and NGAL, with NGAL expression in 33 (26–58) % of the DEFA6-positive cells in inflamed ileum and 49 (19–70) % in control ileum (Fig. 3b). This difference was not significant (*p* = 0.35). There was considerable variance in the intensity and the number of NGAL-stained PCs between individuals, both in controls and active CD. NGAL expression was most prominent in PC granules but there was also staining in the cytoplasm of some cells.

When comparing NGAL expression in the PCs in biopsies from healthy terminal ileum and jejunum (Fig. 3b, c), we found significantly higher expression of NGAL in the PCs in terminal ileum than in the jejunum (*p* = 0.02).

#### Enteroendocrine cells

**Fig. 4:**
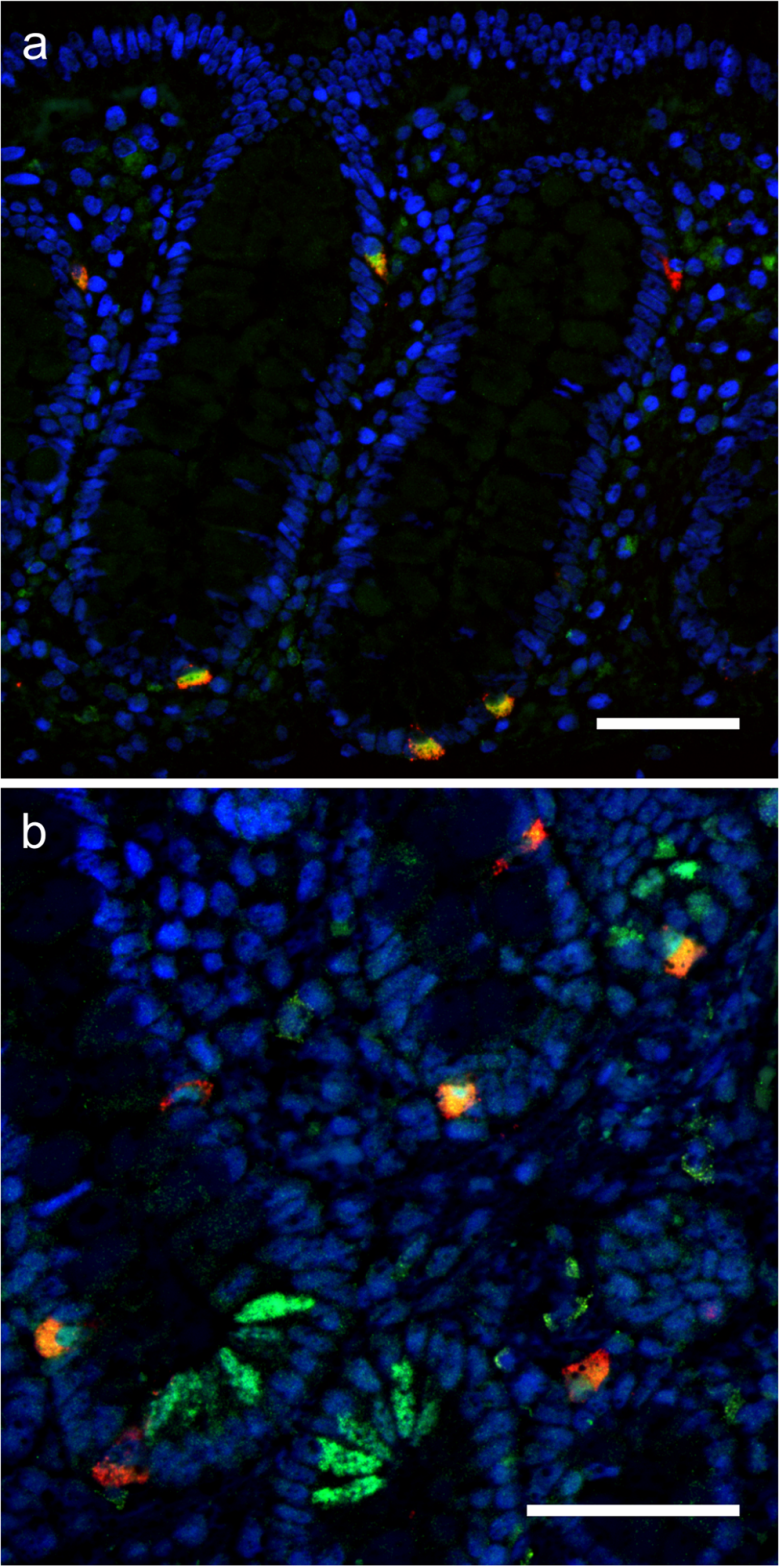
NGAL expression in EECs. Double immunofluorescence. Green: NGAL. Red: CgA. Yellow: overlapping signals. **a** Control colon. Overlapping signals in the majority of EECs. **b** Control ileum. NGAL expression in the majority of EECs. NGAL expression also in PCs. Scale bar 50 μm

In all segments, we noticed staining of cells with a location and morphology consistent with being EECs. Double immunofluorescence staining with CgA confirmed NGAL expression in 63 (49–85) % of CgA-positive EECs in the colon (Fig. 4a) and 53 (45–75) % in the ileum (Fig. 4b), with no difference between controls and active CD (data not shown). The expression in EECs was confirmed with two different antibodies against NGAL. The mouse monoclonal antibody used in the double immunofluorescence staining (ABS-062-14B) showed NGAL in a higher proportion of the EECs than the rabbit polyclonal antibody (ab41105), probably due to lower sensitivity of the latter.

### In situ hybridization confirming immunohistochemistry results

**Fig. 5:**
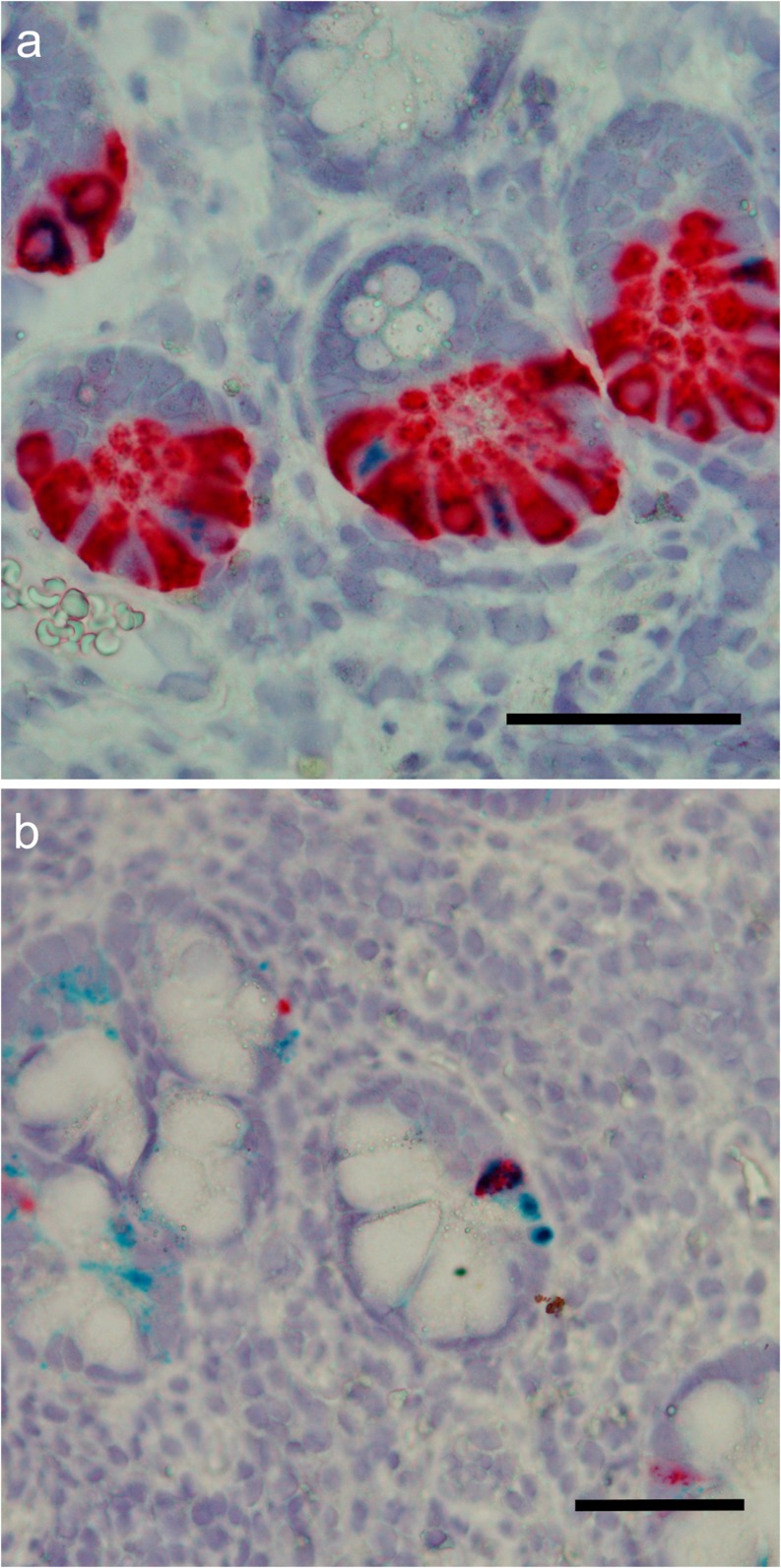
Duplex ISH. **a** Duplex ISH of control terminal ileum showing overlapping signals in a subset of PCs. Red: *DEFA6*. Green: *LCN2.* **b** Duplex ISH of inflamed terminal ileum demonstrating expression of *LCN2* mRNA in EECs. Red: *CHGA*. Green: *LCN2*. Scale bar 50 μm

The *LCN2* ISH signals in PCs were comparable to the NGAL expression pattern seen by IHC (Fig. 5a). ISH confirmed the difference between *LCN2* expression in PCs between healthy terminal ileum and jejunum (*p* = 0.02). ISH also showed *LCN2* expression in EECs, although with generally weaker signals than seen for the NGAL protein. Co-localization was confirmed by duplex ISH with the CgA gene, *CHGA* (Fig. 5b).

## Discussion

We have shown here that *LCN2* is the second most upregulated gene in the mucosa of the terminal ileum in active CD compared to controls, with a 5.86-fold increase. This suggests that NGAL has a major role in the inflammatory process in the ileum, which is a predilection area of CD. In our morphological analyses of ileum, we found that NGAL is expressed in PCs but with no significant difference between active CD and controls. NGAL expression in epithelial cells is, however, clearly increased during inflammation. Although we found marked staining of granulocytes in inflamed biopsies, there is no *LNC2* mRNA in the granulocytes. Bearing in mind the limitations of IHC and ISH as quantitative methods, it is most likely that the massive overexpression of *LCN2* mRNA in our microarray analysis of ileal CD derives from upregulation of this gene and the NGAL protein in epithelial cells other than PCs. One previous study reported NGAL staining of a subset of intestinal PCs in normal individuals (Friedl et al. 1999). The present study is, however, the first to examine NGAL’s expression and regulation in the small intestine in health and disease.

PCs have been suggested to play an important part in the pathogenesis of CD, as several of the genes associated with IBD can be related to these cells. CD risk alleles associated with ATG16L1 and NOD2 can lead to defects in packaging and secretion of antimicrobial peptides in PCs. It has been shown that patients with active CD of the small intestine have a decreased number of PCs, as gene expression of alpha-defensins is reduced during active disease (Perminow et al. 2010; Simms et al. 2008; Wehkamp et al. 2005). Interestingly and in contrast to ileal PCs, the NGAL expression in jejunal PCs is clearly increased during CD inflammation. This discrepancy may be explained by the lower bacterial load in the healthy jejunum compared to the terminal/neoterminal ileum with potentially less baseline stimuli for NGAL induction. The localization of NGAL in the granules along with other antimicrobial peptides suggests a controlled release. The main physiological and pathobiological role of PC NGAL may then be a bacteriostatic effect, with its expression fluctuating due to variable induction by microbial stimuli. This is in contrast to the defensins, lysozyme, Pla2g2a and MMP7, whose expression (in mice) is similar in germ-free and conventional mice (Ouellette 2010).

While PCs are found in the healthy small intestine, these cells are very scarce in the normal colon. However, metaplastic PCs are commonly seen in IBD, both CD and UC. The knowledge of PC’s role in the colon is limited but like in the small intestine, they produce antimicrobial peptides such as lysozyme and defensins and as shown in this study also NGAL. It is also likely that PCs have other role(s), such as providing a niche for neighboring stem cells by secreting Wingless/Int (Wnt) and other factors (Clevers and Bevins 2013).

We moreover show, for the first time, NGAL expression in EECs of the intestine. This cell-type comprises about 1% of the total epithelial cell population and plays an important role in the regulation of appetite and digestive responses through secretion of biogenic amines and peptides. Although the precise role of NGAL in EECs is unknown, recent data show key two-way interactions between EECs and the mucosal immune system (Harrison et al. 2013; Worthington 2015). A completely different role that is consistent with known physiological functions of EECs is also possible, such as suggested in a recent work that proposed NGAL as an appetite suppressing hormone in mice (Mosialou et al. 2017).

In conclusion, we have shown that NGAL is strongly upregulated in active CD of the small intestine, likely due to increased expression in intestinal epithelial cells other than PCs. This upregulation underlines the putative role of NGAL as a fecal biomarker of inflammation in the small intestine. Moreover, we demonstrated the presence of NGAL in PCs and EECs in both healthy and inflamed intestine where its role may be both as an antimicrobial peptide and as a regulatory substance related to the physiological function of EECs.
